# DAPK1 acts as a positive regulator of hypertension via induction of vasoconstriction

**DOI:** 10.1042/CS20255840

**Published:** 2025-06-18

**Authors:** Xiuli Zhang, Ying Cheng, Yao Lu, Nanhui Xu, Zhi Guo, Meizhu Wu, Guosheng Lin, Mengying Yao, Yanyan Yang, Yao Lin, Jun Peng, Aling Shen

**Affiliations:** 1 College of Integrative Medicine, Academy of Integrative Medicine, Fujian University of Traditional Chinese Medicine, Fuzhou, Fujian, China; 2 Fujian Key Laboratory of Integrative Medicine on Geriatrics, Fujian University of Traditional Chinese Med-icine, Fuzhou, Fujian, China; 3 Fujian Collaborative Innovation Center for Integrative Medicine in Prevention and Treatment of Major Chronic Cardiovascular Diseases, Fuzhou, Fujian, China; 4 Department of Cardiology, Xiyuan Hospital of China Academy of Chinese Medical Sciences, Beijing, China; 5 National Clinical Research Center for Cardiovascular Diseases of Traditional Chinese Medicine, Beijing, China; 6 Central Laboratory at the Second Affiliated Hospital of Fujian Traditional Chinese Medical University, Fuzhou, Fujian, China

**Keywords:** DAPK1, hypertension, kinase activity, MLC, vasoconstriction

## Abstract

Death-associated protein kinase 1 (DAPK1) is a tumor suppressor gene involved in apoptosis, autophagy, and tumor progression. However, its role in hypertension (HTN) remains largely unexplored and lacks systematic evaluation. We administered adeno-associated virus (AAV) harboring short hairpin RNA targeting DAPK1 or control short hairpin RNA to male spontaneously hypertensive rats (SHRs) and Wistar-Kyoto rats. Additionally, wildtype and DAPK1 knockout mice were infused with angiotensin II (Ang II) or saline for four weeks. Male C57BL/6 mice underwent a four-week Ang II infusion and were treated with TC-DAPK6, a selective DAPK1 inhibitor. We examined the abdominal aortas (AAs) of mice and rats for pathological changes, measured blood pressure (BP) and pulse wave velocity using noninvasive BP methods, ultrasound, and hematoxylin and eosin staining. The role of DAPK1 in early HTN was further assessed through immunofluorescence, ex vivo isometric constriction of the AA, RNA sequencing, Western blot, and immunohistochemistry. Our study demonstrated that the targeted inhibition of DAPK1 with AAV significantly ameliorated HTN in SHRs and reduced damage to the AAs and target organs, including the heart and kidneys. Meanwhile, DAPK1 knockout or inhibition in mice significantly ameliorates Ang II-induced HTN in mice, as well as reducing damage to the AAs and target organs, including the heart and kidneys. Mechanistically, DAPK1 inhibition prevents myosin light chain (MLC) phosphorylation at serine 19, reducing vasoconstriction and protecting against HTN. In conclusion, DAPK1 is involved in HTN pathogenesis by regulating the MLC pathway to mediate vascular constriction, highlighting potential as a therapeutic target for HTN.

## Introduction

Despite significant advances in clinical screening and therapeutic interventions, hypertension (HTN) remains a prevalent disorder worldwide [1,2], particularly in China, where adults have an HTN prevalence of 27.9% [1]. The cardiovascular system is one of the organs most vulnerable to damage caused by chronic high blood pressure (HBP), which, in turn, causes or worsens cardiovascular conditions [3–6]. Conditions caused by HTN claim the lives of almost 9.4 million people annually [7]. HTN significantly elevates the risk of stroke, heart attack/failure, and kidney disease, thereby increasing mortality and causing a growing strain on healthcare systems [8,9]. Despite treatment with multiple medications, blood pressure (BP) is still poorly governed in many HTN patients, possibly because of additional mechanisms behind HTN that are not addressed by present treatments [10]. Pathological changes in the vasculature have been suggested as contributing factors to HTN development and the following organ damage [11]. Therefore, comprehending the molecular mechanisms behind vascular function is pivotal to developing novel therapies that can reduce cardiovascular risk in HTN patients.

HTN is pathologically characterized by increased vascular contractility [12]. This malfunction of the renin-angiotensin-aldosterone system (RAAS) and excessive stimulation of Angiotensin II (Ang II) are recognized as critical contributors to HTN initiation and advancement [13]. Ang II is a powerful vasoconstrictor and the principal physiologically active element of the RAAS, affecting the functionality and strength of blood arteries by enhancing vascular smooth muscle cells (VSMCs) contractile activity and proliferation [14–16]. In VSMCs, Ang II binding to angiotensin type 1 receptor (AT1R) triggers phospholipase C activation, thereby increasing cytoplasmic calcium (Ca^2+^) concentration and leading to VSMC contraction, rapid vasoconstriction, and HBP [17,18]. Up-regulated Ca^2+^ levels activate kinases such as calmodulin-dependent kinase II (CaMK II) and myosin light chain (MLC) kinase, which promotes vascular contraction through MLC phosphorylation [19–21]. The altered regulation of these cellular signaling components may result in a prolonged vasoconstrictive condition and persistent HTN. Consequently, targeting calcium-related signaling pathways in VSMCs may constitute an intriguing treatment option for HBP.

Death-associated protein kinase (DAPK), a Ca^2+^/calmodulin (CaM)-dependent serine/threonine kinase, mainly contributes to cell death [22]. The DAPK family comprises DAPK1/2/3 and the apoptosis-inducing kinases DRAK1/2 [23]. Previous studies have demonstrated increased DAPK3 expression in spontaneously hypertensive rats (SHRs), suggesting its potential involvement in HTN through mechanisms such as vascular inflammation, vascular remodeling, and related pathways [23–25]. Nonetheless, the specific implication of DAPK1 in HTN and the mechanisms behind it remain unexplored. Therefore, we aimed to reveal the impact of DAPK1 on HTN.

## Methods

### Reagents

Ang II was obtained from Abcam (Cambridge Science Park, Cambridge, UK, ab120183), while procuring the protein assay, trypsin, EDTA (25200072), fetal bovine serum (FBS; 10091148), and DMEM (C11995500BT) from Thermo Fisher Scientific (Waltham, MA, U.S.A.). Signalway Antibody Limited Liability Company(Greenbelt, MD, U.S.A.) supplied antibody #11114, which is specific to MLC (Phospho-Ser19). The Glyceraldehyde-3-Phosphate Dehydrogenase(GAPDH) antibody (Abp57259) was sourced from Abbkine Scientific (Wuhan, Hubei, China). Cell Signaling Technology of Danvers (MA, U.S.A.) supplied the rabbit secondary antibody (7074) and the antibody against MLC (3672 s). Maixin Biotechnology (Fuzhou, Fujian, China) provided the used products, as well as the antigen repair solution, Ultra-Sensitive™ SP (Mouse/Rabbit) IHC kit, and DAB kit. The polyvinylidene difluoride(PVDF) membranes (IPVH00010) were procured from Millipore (Billerica, MA, U.S.A.).

### Animals

All animals were anesthetized using 2% isoflurane (in 100% oxygen) delivered via a precision vaporizer and induction chamber. The depth of anesthesia was monitored by assessing the absence of pedal withdrawal reflex and respiratory rate. Post experiment, animals were killed by cervical dislocation under deep anesthesia. Experiments followed the Animal Research: Reporting of In Vivo Experiments (ARRIVE) guidelines and the Animal Use Committee of Fujian University of Traditional Chinese Medicine (FJTCMIACUC 2021060, 2022083, 2022097). The male SHRs and Wistar Kyoto (WKY) rats (four weeks old) were sourced from Beijing Vital River Laboratory Animal Technology Co., Ltd. (Beijing, China). The Mutant Mouse Resource & Research Centers (MMRRC) at UC Davis, which is financed by the National Institutes of Health (NIH), provided the DAPK1 knockout transgenic mice that were used in this study (RRID: MMRRC_037822-UCD). The male C57BL/6 mice (8–10 weeks old) were procured from Laboratory Animal Technology in Shanghai, China. Beijing Huafukang Bioscience CO., Ltd. (Beijing, China) provided adult male Wistar rats (6–8 weeks old). Under controlled conditions free of specific pathogens, all models were kept in a 12-h light–dark cycle at 24±2°C and 50–60% humidity. The models were allowed unlimited access to nourishment and drinking water during the trial.

### Construction of DAPK1 knockdown, knockout, and inhibitor model

For knockdown experiments, male SHRs and WKY rats (age 4 weeks) were subcutaneously injected with an adeno-associated virus (AAV) encoding a short hairpin RNA targeting rat death-associated protein kinase 1 (DAPK1) (sh-DAPK1) or a control short hairpin RNA (sh-Control) in sterile saline (1×10^13^ viral genomes/animal). A total of 14 male WKY rats and 14 male SHR were allocated at random into the following groups: WKY+sh-Control, WKY+sh-DAPK1, SHR+sh-Control, SHR+sh-DAPK1 (*n*=7 for each group). The Ethical Code is FJTCMIACUC 2021060.

For knockout experiments, male DAPK1 knockout mice and their littermate controls at 8–10 weeks were allocated at random into four groups: DAPK1^+/+^ + Saline, DAPK1^+/+^ + Ang II, DAPK1^−/−^ + Saline, and DAPK1^−/−^ + Ang II (*n*=5 for each group). Both DAPK1^+/+^ + Ang II and DAPK1^−/−^ + Ang II groups were subjected to infusion with Ang II (500 ng/kg/min), whereas both DAPK1^+/+^ + Saline and DAPK1^−/−^ + Saline groups were infused with saline for one month via subcutaneously implanted micro-osmotic pumps. The ethical code is FJTCM IACUC 2019047.

A total of 24 male C57BL/6 mice were allocated at random to one of the four groups (*n*=6 for each group): Control, Ang II, Ang II + TC-DAPK6-L, or Ang II + TC-DAPK6-H, in order to conduct DAPK1 inhibitor tests. The mice that were part of the Ang II + TC-DAPK6-L and Ang II + TC-DAPK6-H groups were given Ang II (500 ng/kg/min rate) and TC-DAPK6 (1 or 10 mg/kg/day rate), respectively, administered intraperitoneally (200 μl for each mouse in each group). The control group received saline and Ang II at 500 ng/kg/min for each via subcutaneously implanted micro-osmotic pumps, as well as given equal volumes of saline intraperitoneally for four weeks. Code for ethical considerations: FJTCMIACUC 2022097.

### Measurement of BP in animals

BP was measured by the noninvasive tail-vein BP instrument (Kent Scientific; Torrington, CT, U.S.A.) by first placing animals in a heating chamber at 35°C, stabilized for 5–10 min, and was followed by 5 pre-cycles. Systolic/diastolic blood pressure [(SBP)/(DBP)] and mean arterial pressure (MAP) were averaged from ten measurements for each mouse.

### Ultrasound in animals

At the conclusion of the studies, all animals were anesthetized using 2% isoflurane and placed on a pre-warmed (37°C) platform. The heart rate was sustained at 300–350 beats/min for rats and approximately 450 beats/min for mice using 1.5% isoflurane. The pulse wave velocity (PWV) and abdominal aorta (AA) thickness were measured by the Vevo 2100 ultrasound equipment (VisualSonics; Toronto, Ontario, Canada). In B mode, the AA under the sternum and the xiphoid processes were imaged using an ultrasonic probe operating at 20 MHz in rats and 30 MHz in mice. In M mode, there were distal and proximal time delays of blood vessels. Using the Vevo® LAB software (VisualSonics; Toronto, Ontario, Canada), the PWV and thickness of the AA were computed.

### Hematoxylin-eosin (H&E) staining

At the conclusion of the trial, the experimental models were sedated with isoflurane and subsequently executed. The AA, heart, and kidney tissues were harvested and preserved in 4% paraformaldehyde for 48 h at room temperature (RT). Thereafter, the sections were dehydrated via an alcohol gradient, embedded in paraffin, sectioned into 4-μm-thick slices, dewaxed, and stained with H&E at RT. The stained tissue sections were photographed utilizing an automated optical microscope (Leica DM4000B; Wetzlar, Germany) at a magnification of 400×.

### RNA-sequencing analysis

The objective of the RNA sequencing was to detect enriched pathways and differentially expressed transcripts (DETs). After slicing the AA, it was maintained in RNAlater for 24 h at RT before being frozen at −20°C until analysis. Capital Bio-Technology of Beijing, China, was entrusted with the RNA-sequencing task. Basically, RNA-sequencing library building and polymerase chain reaction (PCR) amplification, as well as total RNA isolation and purification, were the subsequent steps. The samples were obtained from tissues within the AA. Libraries were prepared by the NEBNext Ultra RNA Library Kit (Illumina, Ipswich, MA, U.S.A.) per protocols. RNA quantity and integrity were ascertained with Qubit 3.0 and Agilent 2100 Bioanalyzers. DETs were identified using DESeq (v1.28.0) with |fold change| ≥ 2 and *P*<0.05. The DETs were subsequently examined in relation to the data acquired from the Gene Ontology (GO) and Kyoto Encyclopedia of Genes and Genomes(KEGG) databases.

### Immunohistochemical (IHC) analysis

The AA tissues were sectioned into 4-μm-thick slices for IHC labeling. The sections were rehydrated employing gradient ethanol doses, fixed in a 0.01-M citrate antigen repair solution for 10 min at 100°C, and cooled down to RT. The tissue sections were subsequently treated overnight at 4°C with primary antibodies targeting DAPK1 (1:200), p-DAPK1 (S308) (1:100), MLC (1:200), and p-MLC (S19) (1:200). Subsequent to three washes with phosphate buffered saline(PBS). The sections were treated with a secondary antibody for 1 h, subsequently followed by conjugated horseradish peroxidase-labeled streptavidin. The sections subsequently underwent incubation with diaminobenzidine as the chromogen, following the manufacturer’s guidelines. Images were acquired at 400× magnification utilizing an automated optical microscope (DM4000B; Leica). The proportion of positively stained cells was assessed utilizing Image J software (NIH, MD, U.S.A.).

### Isometric contraction of AA *ex vivo*

Male DAPK1^+/+^ and DAPK1^−/−^ mice (*n*=6) were subjected to anesthesia with 2% isoflurane and killed; then, the AA was quickly dissected and sliced into approximately 3-mm-long ring segments in cold physiological saline solution (PSS) that contained 130 mM NaCl, 4.7 mM KCl, 1.18 mM KH_2_PO_4_, 1.17 mM MgSO_4_, 14.9 mM NaHCO_3_, 5.5 mM glucose, 0.026 mM EDTA, and 1.16 mM CaCl_2_, with pH adjusted to 7.4 using 3 M NaOH. These rings were sustained in an organ chamber filled with 5 ml PSS with a gas mixture (95% O_2_ and 5% CO_2_) at 37°C, which were measured at a resting tension of approximately 2 mM using a force transducer (Danish Myo Technology; Radnoti, Monrovia, CA, U.S.A.) [26]. Each AA ring was stabilized for 45 min and then stimulated with KCl (60 mM) to observe the reactivity. The AA rings were stimulated with Ang II (1 μM) for the measurement of relaxation rates.

### VSMCs isolation and identification

Primary VSMCs were isolated from rats AA by enzymatic digestion [27] and cultured in DMEM supplemented with 10% FBS and 1% penicillin/streptomycin (100 IU/ml; 100 μg/ml) at 37°C with 95% air and 5% CO_2_. Primary VSMCs were identified by their substantial α-SMA expression (a VSMC marker). Cells (passages 2–6) were randomly assigned to treatment groups based on the experimental design (Ethical code: FJTCM IACUC 2022083).

### Western blot analysis

Before being centrifuged at 12,000 *g* for 20 min at 4°C, the cells were lysed in a Western & IP cell lysis solution that also contained phenylmethanesulfonyl fluoride, cocktail, and PhosStop. Using a BCA kit, the concentration of proteins was ascertained. After being separated on SDS-PAGE, the same amount of protein was transferred to PVDF membranes, blocked with 5% non-fat milk in tris buffered saline with tween (TBST, TBS+0.1% Tween) at 37°C for 2 h at RT. The membranes were incubated for a whole night at 4°C with primary antibodies against DAPK1, p-DAPK1(S308), MLC, and p-MLC(S19) (all 1:1000), or GAPDH (1:5000). After three TBST washes, they were incubated with a secondary rabbit antibody (1:5000) for 2 h at RT and detected using enhanced chemiluminescence (Amersham ImageQuant 800, Cytiva, U.S.A.). Optical density was analyzed with ImageJ.

### Rhodamine phalloidin staining

Following the protocols, the cytoskeletal organization was assessed using phalloidin staining. The VSMCs were placed into confocal dishes, grown for one night, and then induced with Ang II (1 μM) either with or without a DAPK1 inhibitor (TC-DAPK6). Thereafter, cells were fixed with 4% paraformaldehyde for 30 min and stained with rhodamine phalloidin (AAT Bioquest; Sunnyvale, CA, U.S.A.) at a final concentration of 100 nM for 30 min. After 20 min, the nucleus was stained with DAPI. At a magnification of 200×, the stained cells were examined using a fluorescent microscope (PerkinElmer, CA, U.S.A.).

### Statistical analysis

Analyses were conducted using GraphPad Prism 8.0 (GraphPad Software, https://www.graphpad.com) and SPSS 25.0 (IBM, https://www.ibm.com/analytics/spss-statistics-software), reporting the data as mean ± SD. An unpaired Student’s *t*-test compared two groups (Bonferroni for chi-square variance, Games-Howell otherwise). One-way analysis of variance (ANOVA) was used to analyze three or more groups with similar post hoc tests. Two-way ANOVA with repeated measures assessed group differences. A *p-*value *<*0.05 indicated statistical significance.

## Results

### DAPK1 knockdown down-regulated BP, vascular and target organ damage in SHRs

**Figure 1:**
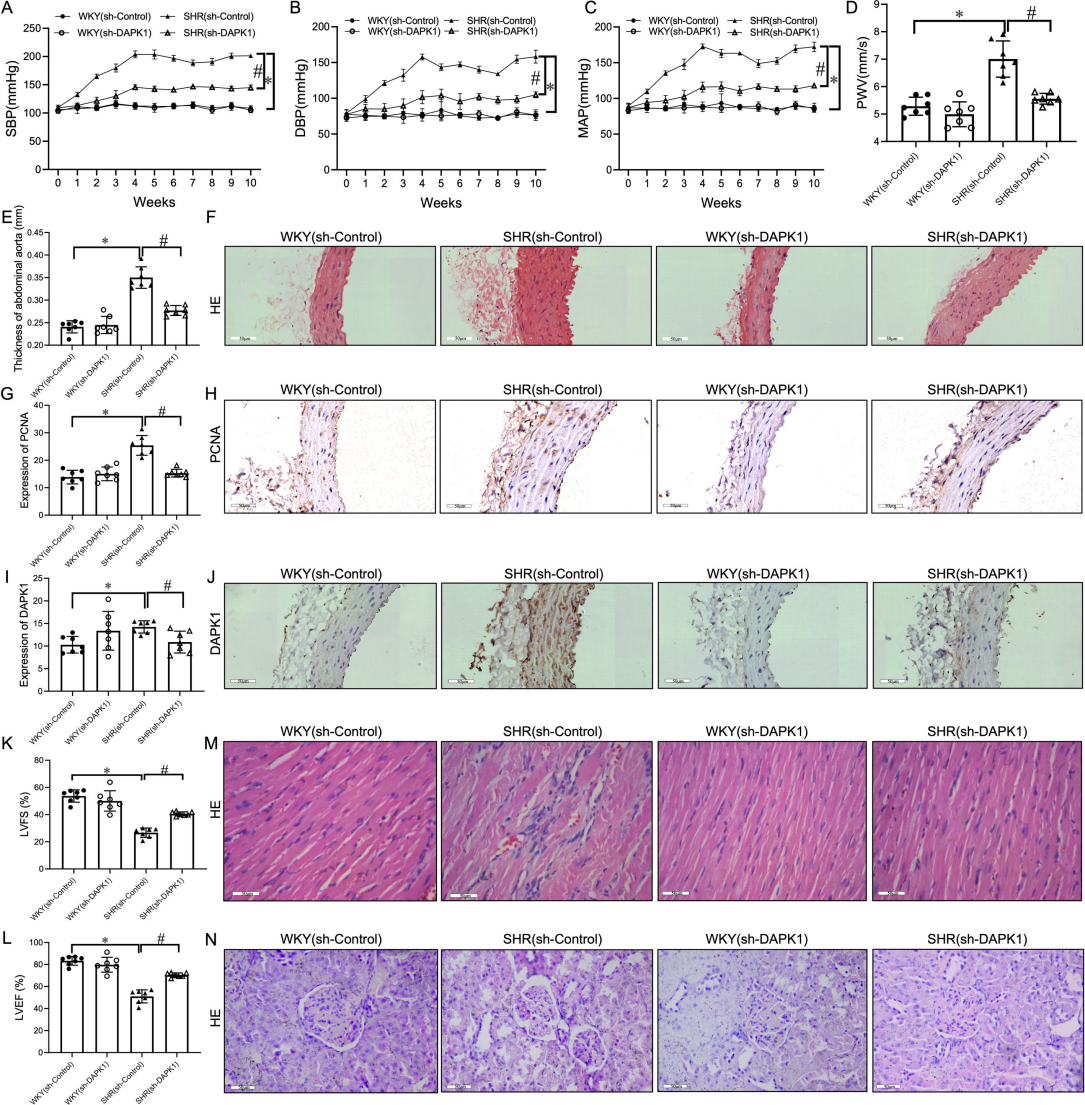
Death-associated protein kinase 1 (DAPK1) suppression lowers blood pressure and reduces vascular and organ damage in spontaneously hypertensive rats (SHRs). (**A–C**) Tail-cuff plethysmography: systolic blood pressure (SBP), diastolic blood pressure (DBP), and mean arterial pressure (MAP). (**D,E**) Ultrasound: pulse wave velocity (PWV) and aortic wall thickness. (**F**) Hematoxylin-eosin (H&E)-stained abdominal aorta sections (400× magnification). (**G–J**) immunohistochemical (IHC) analysis: proliferating cell nuclear antigen (PCNA) (**G,H**) and DAPK1 (**I,J**) expression in aortic tissues (scale bar = 50 µm). (**K,L**) Echocardiography: left ventricular fractional shortening (LVFS) and left ventricular ejection fraction (LVEF). (**M,N**) H&E-stained cardiac and renal tissues (scale bar = 50 µm). *n*=7, ^*,#^*p*<0.05 vs. sh-Control and sh-DAPK1, respectively.

To confirm the DAPK1 function in HTN, negative control and DAPK1 knocked-down (KD), AAV was constructed and injected into WKY and SHR through the tail vein to construct a control group (sh-Control) and DAPK1 KD (sh-DAPK1) WKY and SHR models. The findings indicated a significant decrease in SBP, DBP, and MAP (Figure 1A–C) in SHR (sh-DAPK1) compared with SHR (sh-Control). Notably, the results revealed no significant variation in body weight between each group (Supplementary Figure S1A). Furthermore, ultrasound and histopathological analyses showed that DAPK1 knockdown significantly down-regulated PWV (Figure 1D) and AA thickness (Supplementary Figure S1B,E and F). The IHC staining further demonstrated that knockdown of DAPK1 down-regulated the protein expression of proliferating cell nuclear antigen (PCNA) (Figure 1G and H) and DAPK1 protein (Figure 1I and J) in SHR.

Moreover, echocardiography revealed that DAPK1 suppression markedly reversed cardiac contractile dysfunction, as indicated by increased left ventricular fractional shortening (LVFS) and left ventricular ejection fraction (LVEF) compared with SHR (sh-Control) (Supplementary Figure S1C, K and L). DAPK1 knockdown also attenuated cardiac histologic abnormalities, including disordered myocardial fibers and inflammatory cell infiltration (Figure 1M). Similarly, H&E staining revealed that DAPK1 inhibition significantly alleviated pathological changes in renal tissue, including glomerular atrophy, epithelium atrophy, and tubular dilatation, compared with SHR (sh-Control) (Figure 1N).

### DAPK1 deficiency ameliorated Ang II-induced HTN, vascular and target organ pathologies

**Figure 2:**
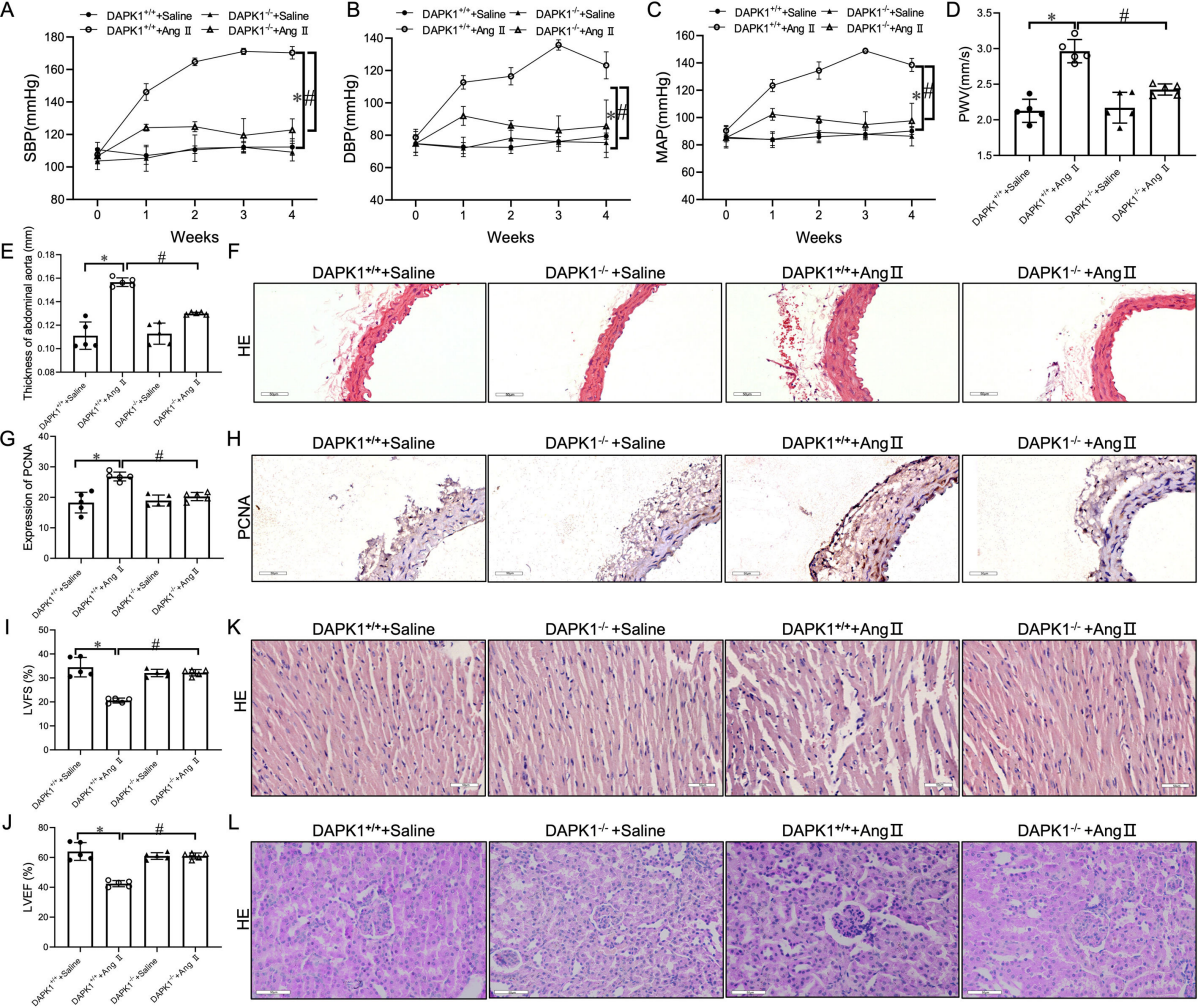
DAPK1 deficiency alleviates angiotensin II (Ang II)-induced hypertension and vascular pathology. (**A–C**) SBP, DBP, and MAP in each group. (**D,E**) Ultrasonography: PWV and aortic wall thickness. (**F**) H&E-stained cross-sections of abdominal aorta tissues (scale bar = 50 µm). (**G,H**) IHC analysis: PCNA expression in aortic tissues (scale bar = 50 µm). (**I,J**) LVFS and LVEF. (**K,L**) H&E-stained cardiac and renal tissues (scale bar = 50 µm). *n*=5, *^,#^*p*<0.05 vs. DAPK1^+/+^ + Ang II and DAPK1^−/−^ +Ang II, respectiely.

To ascertain the DAPK1 impact on HTN, we compared the BP of wild-type (DAPK1^+/+^) and DAPK1 knockout (DAPK1^−/−^) mice post-Ang II infusion for 28 days using the noninvasive tail-cuff method. In comparison with the DAPK1^+/+^ + Ang II group, Ang II infusion failed to increase SBP, DBP, and MAP (Figure 2A–C) in DAPK1^−/−^ mice, with no significant alteration in body weight between DAPK1^+/+^ + Ang II and DAPK1^−/−^ + Ang II groups (Supplementary Figure S2A). Ultrasound and H&E staining showed that Ang II infusion significantly escalated arterial PWV and AA thickness of DAPK1^+/+^ mice, whereas DAPK1 knockout impeded these alterations, implying that DAPK1 deficiency attenuated Ang II-provoked vascular injury (Supplementary Figure S2B, D–F). Meanwhile, PCNA expression was significantly decreased in AA tissues of Ang II-treated DAPK1^−/−^ mice, unlike the DAPK1^+/+^ mice (Figure 2G and H).

Moreover, echocardiography revealed that DAPK1^−/−^ markedly reversed Ang II-induced cardiac dysfunction by decreasing LVFS and LVEF compared with DAPK1^+/+^ mice (Supplementary Figure S2C, I and J). Then, we next examined the pathological changes in the target organ, including the heart and kidney. Figure 2K depicts that DAPK1^−/−^ attenuated Ang II-induced cardiac myocardial fiber disorder and inflammatory cell infiltration in cardiac tissues compared with DAPK1^+/+^ mice. Similarly, H&E staining showed Ang II-induced glomerular atrophy and tubular dilatation in the renal tissues of DAPK1^+/+^ mice, which DAPK1 knockout mitigated (Figure 2L).

### DAPK1 deficiency attenuated Ang II-induced constriction in AA tissues of hypertensive mice

**Figure 3:**
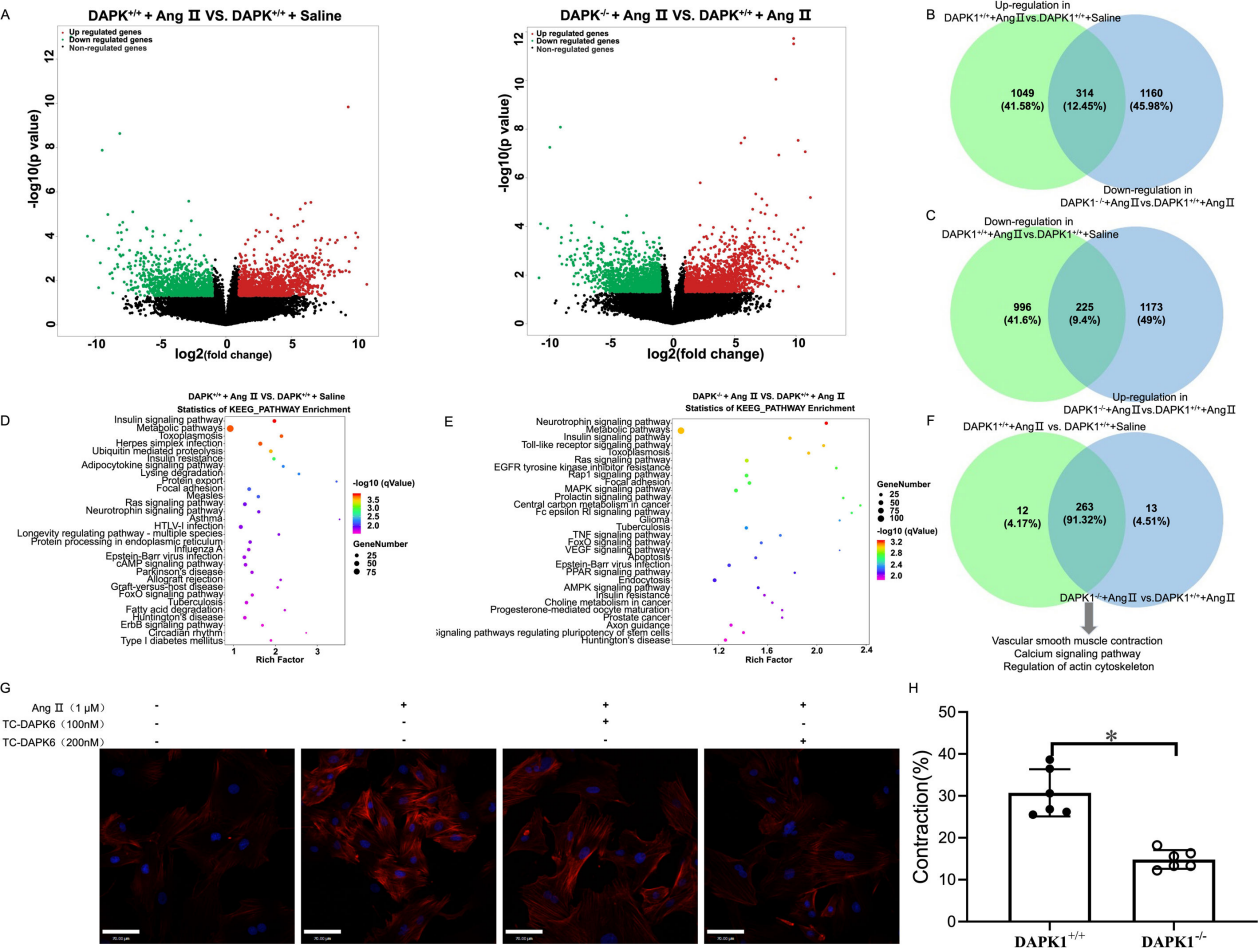
DAPK1 deficiency mitigates Ang II-provoked aortic constriction in hypertensive mice. (**A**) Volcano plots: differentially expressed transcripts (DETs) in abdominal aorta tissues (*p*<0.05, |fold change| ≥ 2). (**B,C**) Comparative transcript analysis: increased and decreased DETs in DAPK1^+/+^+ Ang II vs. DAPK1^+/+^ + saline groups and DAPK1^−/−^ + Ang II vs. DAPK1^+/+^ + Ang II. (**D–F**) KEGG analysis: Top 30 enriched pathways in DAPK1^+/+^ + Ang II vs. DAPK1^+/+^ + saline (**D**) and DAPK1^−/−^ + Ang II vs. DAPK1^+/+^ + Ang II (**E**), with integrated pathway overlaps (**F**). (**G**) Immunofluorescence staining: F-actin (red) with DAPI-labeled nuclei (blue); scale bar = 20 µm. (**H**) Effect of DAPK1 knockdown on tension in Ang II pre-constricted abdominal aorta rings.

Subsequently, we sequenced the RNA of the AA of DAPK1^+/+^ and DAPK1^−/−^ mice that were injected with Ang II to better understand the transcriptional alterations and the molecular mechanisms that underlie DAPK1’s protective actions in a model of HTN in mice. In DAPK1^+/+^ + saline mice, the cluster maps (Supplementary Figure S3A**, left panel**) and volcano maps (Figure 3A**, left panel**) revealed 1363 increased transcripts and 1221 decreased transcripts compared with DAPK1^+/+^ + Ang II mice. In DAPK1^+/+^ + Ang II mice, the cluster maps (Supplementary Figure S3A**, right panel**) and volcano maps (Figure 3A**, right panel**) revealed that 1398 DETs were increased, whereas 1474 DETs were down-regulated compared with DAPK1^−/−^ + Ang II mice. The integrated comparative analysis between DAPK1^+/+^ + Ang II vs. DAPK1^+/+^ + saline and DAPK1^−/−^ + Ang II vs. DAPK1^+/+^ + Ang II groups identified 314 of the 1363 up-regulated transcripts in the DAPK1^+/+^ + Ang II group that were diminished in the DAPK1^−/−^ + Ang II group (Figure 3B), while 225 of 1221 repressed transcripts in the DAPK1^+/+^ + Ang II group were overexpressed after DAPK1 knockout (Figure 3C).

The compared DETs of the DAPK1^+/+^ + saline and DAPK1^+/+^ + Ang II groups revealed enrichment of many KEGG pathways into various signaling pathways, including cyclic adenosine monophosphatec(AMP), forkhead box O(FoxO), and focal adhesion signaling (Figure 3D). Additionally, mitogen-activated protein kinase(MAPK), tumor necrosis factor(TNF), and AMP-activated protein kinase(AMPK) signalings were enhanced in the DETs of the DAPK1^+/+^ + Ang II and DAPK1^−/−^ + Ang II groups, respectively (Figure 3E). The comparative integrated analysis DAPK1^+/+^ + Ang II vs. DAPK1^+/+^ + saline and DAPK1^−/−^ + Ang II vs. DAPK1^+/+^ + Ang II showcased that vascular smooth muscle contraction, calcium signaling pathway, and actin cytoskeleton regulation were enriched (Figure 3F). Based on RNA sequencing results, we further explored the impacts of DAPK1 on Ang II-provoked vascular smooth muscle cytoskeleton and vasoconstriction. Phalloidin staining showed that TC-DAPK6 (a potent kinase activity inhibitor of DAPK1) treatment alleviated the formation of actin stress fibers in Ang II-stimulated VSMCs (Figure 3G). The present research demonstrated the effect of DAPK1 on vasoconstriction in isolated aortic rings from the AA of DAPK1^+/+^ and DAPK1^−/−^ mice. The results showed that DAPK1 knockout significantly down-regulated constriction in AA rings contracted with Ang II (Figure 3H).

### DAPK1 inhibitor (TC-DAPK6) reversed Ang II-induced HTN, vascular and target organ pathologies

**Figure 4:**
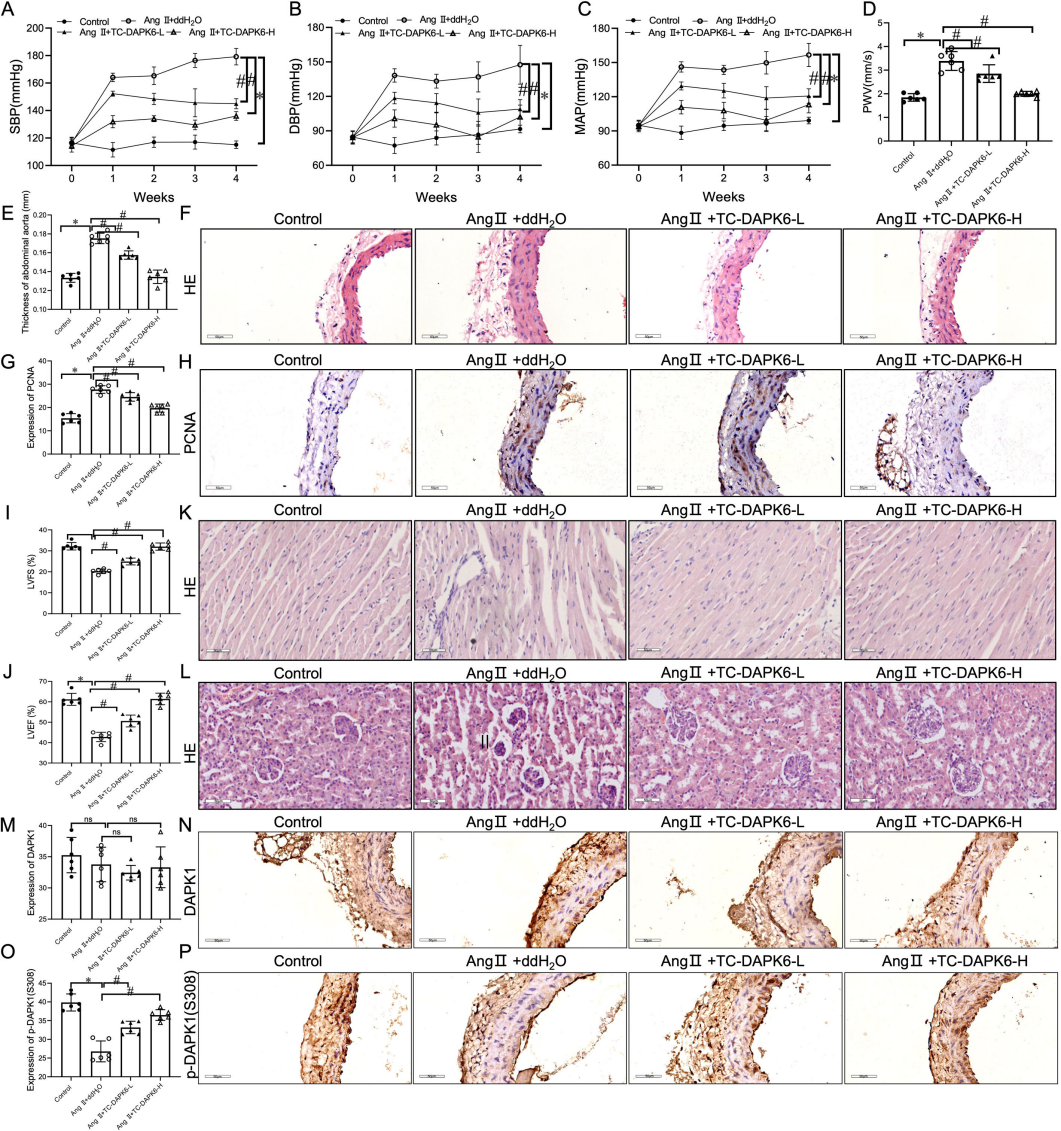
DAPK1 inhibition reverses Ang II-induced hypertension and vascular damage. (**A–C**) TC-DAPK6 reduces SBP, DBP, and MAP in Ang II-triggered hypertensive mice. (**D,E**) Ultrasonography: PWV and aortic wall thickening. (**F**) H&E-stained cross-sections of the abdominal aorta. (**G,H**) IHC analysis: PCNA expression in aortic tissues (scale bar = 50 µm). (**I,J**) LVFS and LVEF measurements. (**K,L**) H&E-stained cardiac and renal tissues. (**M–P**) IHC analysis: DAPK1 (**M**) and p-DAPK1(S308) (**O**) expression in aortic tissues, with statistical representation. *n*=6,*^,#^*p*<0.05 vs. Control and Ang II + ddH₂O, respectively.

Because of the critical roles of Ang II in HTN and vascular diseases, we investigated whether DAPK1 kinase activity is regulated in Ang II-infused hypertensive mice. Herein, we applied TC-DAPK6 to intervene in Ang II-triggered hypertensive mice to further explore the impact of inhibition of DAPK1 kinase activity on HTN. The BP measurements revealed an increase in SBP, DBP, and MAP in Ang II-infused mice relative to control mice, which were attenuated following treatment with various TC-DAPK6 concentrations (1 or 10 g/kg/day; Figure 4A–C). The results manifested no significant variance in body weight between each group (Supplementary Figure S4A). Moreover, in comparison with the control group, the up-regulation of PWV (Figure 4D), aortic wall thickness (Supplementary Figure S4B, E and F), and the expression of PCNA (Figure 4G and H) in the AAs of Ang II-infused mice were alleviated after treatment with various TC-DAPK6 concentrations (Figure 4D–H).

To investigate the cardiac protective effect of DAPK1, we compared the cardiac function across groups using echocardiography. Figure 4I and J present that LVFS and LVEF were significantly diminished in the Ang II group in contrast with the control (Supplementary Figure S4C, I and J). However, TC-DAPK6 treatment attenuated the decreases in LVFS and LVEF (Figure 4I and J). Moreover, Ang II-provoked cardiac myocyte hypertrophy, myocardial fibers disorder, and inflammatory cell infiltration were attenuated in the TC-DAPK6 group compared with Ang II group mice after 4 weeks of treatment (Figure 4K). Additionally, TC-DAPK6 intervention alleviated Ang II-induced renal pathological changes in hypertensive mice (Figure 4L).

Importantly, DAPK1 expression was not affected in the AA of Ang II-infused mice (Figure 4M and N), but p-DAPK (S308) expression was significantly down-regulated. However, the TC-DAPK6 treatment significantly suppresses the decrease in p-DAPK (S308) expression (Figure 4O and P).

### Ang II stimulation promoted the increase in intracellular calcium concentration and DAPK1 kinase activity in VSMCs

**Figure 5:**
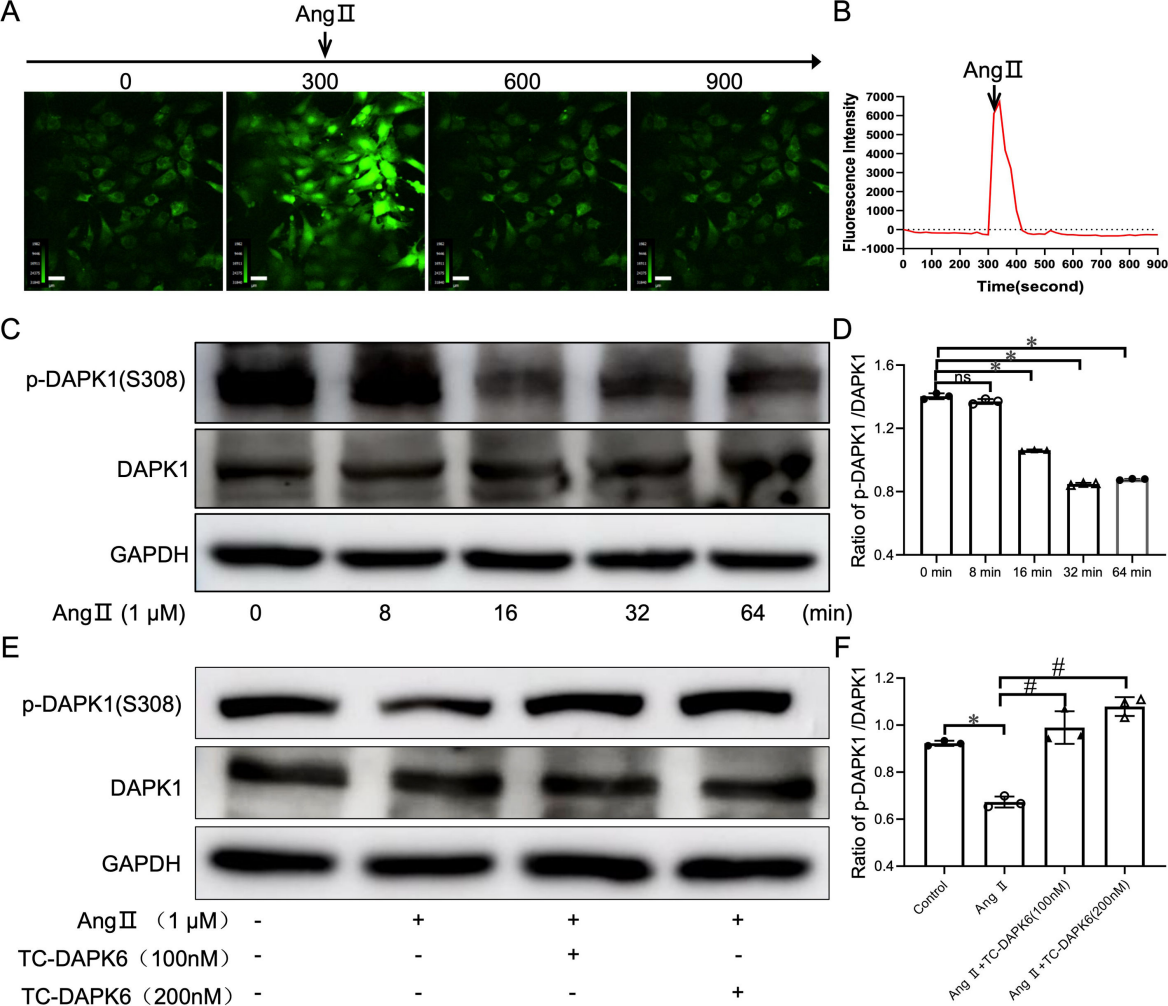
Ang II increases intracellular calcium and activates DAPK1 in vascular smooth muscle cells (VSMCs). (**A,B**) Real-time confocal microscopy: VSMCs loaded with Fluo-4 showing intracellular Ca²^+^ increase after Ang II (1 μM) stimulation (200× magnification). Ca²^+^ levels were normalized to baseline. (**C,D**) Western blotting: p-DAPK1(S308)/DAPK1 ratio over time (*P<0.05 vs. 0 min). (**E,F**) Western blot analysis was performed to determine the ratio protein expression of p-DAPK1(S308)/DAPK1 protein expression, *p<0.05 vs. the Control group, #*p*<0.05 vs. Ang II + group.

To confirm the role of Ca^2+^ on DAPK1 signaling pathway activation *in vitro*, based on RNA sequencing results, primary VSMCs were isolated and identified by being stained with α-SMA (Supplementary Figure S5A). The release of intracellular Ca^2+^ in VSMCs was observed using a double turntable laser confocal microscope after labeling with fluo-4/AM, showing that Ang II stimulation promotes intracellular Ca^2+^ release (Figure 5A and B). Subsequent *in vitro* determination of DAPK1 activation showed a decrease in the ratio of (p-DAPK1 (S308))/(DAPK1) in Ang II-triggered VSMCs (Figure 5C and D), indicating that Ang II activates DAPK1. Furthermore, Western blotting showed that the ratio of p-DAPK1 (S308)/(DAPK1) significantly decreased after Ang II stimulation of VSMCs for 32 min, whereas these levels increased after treatment with various concentrations of TC-DAPK6 (Figure 5E and F). The results showed that Ang II stimulation increases intracellular Ca^2+^ concentration, which subsequently activates DAPK1 kinase activity.

### DAPK1 lowered BP by inhibiting MLC-mediated vasoconstriction

**Figure 6:**
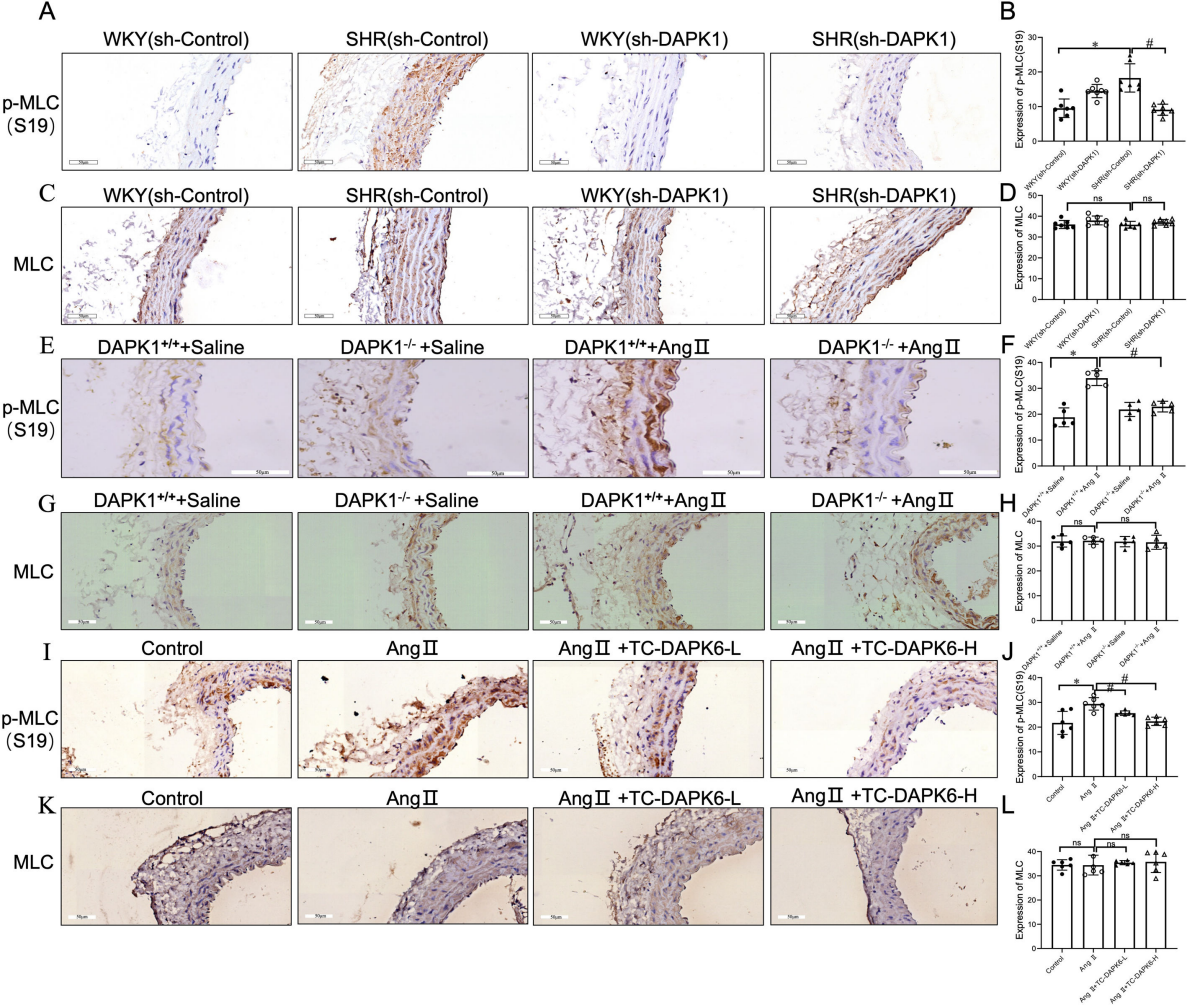
DAPK1 lowers blood pressure by inhibiting myosin light chain (MLC)-mediated vasoconstriction. IHC analysis: Phosphorylated MLC at Ser19 (p-MLC(S19)) and total MLC in abdominal aortic tissues. (**A–D**) p-MLC(S19) and MLC levels in SHR (*n*=7, *^,#^*p*<0.05 vs. sh-Control and sh-DAPK1, respectively). (**E–H**) DAPK1^+/+^ and DAPK1^−/−^ mice treated with Ang II (*n*=5, *^,#^*p*<0.05 vs. DAPK1^+/+^ + Ang II and DAPK1^−/−^ + Ang II, respectively). (**I–L**) Control vs. Ang II + ddH₂O (*n*=6, *^,#^*p*<0.05 vs. Control and Ang II, respectively); 400× magnification, scale bar = 50 μm.

Ca^2+^ promotes the dephosphorylation and activation of the serine 308 site of DAPK1, which, in turn, catalyzes MLC phosphorylation at serine 19, enhancing myosin contractility [28]. To explore this mechanism, we further determined the phosphorylation level of MLC, a key gene in the calcium signaling pathway. The IHC staining demonstrated that p-MLC (S19) protein levels were significantly down-regulated in the SHR (sh-DAPK1) group compared with the SHR (sh-Control) group (Figure 6A and B), while total MLC levels remained unaffected between each group (Figure 6C and D). Similarly, DAPK1 knockout significantly attenuated the up-regulation of p-MLC (S19) in the AA of Ang II-induced hypertensive mice (Figure 6E and F), without affecting total MLC levels (Figure 6G and H). Furthermore, the DAPK1 inhibitor TC-DAPK6 also significantly attenuated the increase in p-MLC (S19) in the AA of Ang II-induced hypertensive mice (Figure 6I and J). There was no significant variation in MLC expression between each group (Figure 6K and L). Additionally, Western blotting indicated that treatment with various concentrations of TC-DAPK6 significantly reduced the up-regulation of p-MLC (S19)/MLC ratio in Ang II stimulated VSMCs (Supplementary Figure S6A and B). Collectively, DAPK1 is crucial in Ang II-provoked vasoconstriction by phosphorylating MLC.

## Discussion

In the present investigation, we found that DAPK1 in VSMCs regulates BP and the elastic characteristics of the AA. Mechanically, we predicted and verified that DAPK1 reduced BP and attenuated HTN-induced vascular dysfunction, partly by inhibiting MLC pathway activation. These findings were validated through RNA sequencing and validated by *in vivo*, *ex vivo*, and *in vitro* experiments. Furthermore, DAPK1 signaling in VSMCs is crucial in Ang II-provoked HTN. A chronic elevation in circulating Ang II led to dephosphorylation of p-DAPK1(308), thereby activating the DAPK1 signaling pathway, which results in vasoconstriction and HTN.

The World Health Organization reported that over 30% (1.38 billion) of the global elderly population is afflicted with HTN [2], a significant preventable risk factor for cardiovascular disease and the major mortality driver globally. Consequently, a comprehensive understanding of the underlying mechanisms is crucial for the prevention and treatment of HTN [29]. As an integral component of the death protein kinase family, DAPK1 is related to DAPK2/3, the closest relatives of DAPK1, which have 83–80% amino acid identity with DAPK1’s kinase domain, correspondingly [30–33]. Previous studies have found that DAPK3 can participate in the regulation of HTN [24,25]. Therefore, we hypothesize that DAPK1 could be a potential novel target for the regulation of HTN.

DAPK1 was initially discovered through a screening process to identify genes that affect γ-interferon-induced HeLa cell death [34]. Herein, DAPK1 was elevated in the AA of SHRs, which did not affect DAPK1 expression in the AA of Ang II-triggered hypertensive mice and in Ang II-stimulated VSMCs. However, the p-DAPK (S308) expression was down-regulated. DAPK1 is controlled through numerous pathways. First, binding to Ca^2+^-activated CaM activates DAPK1 by relieving the auto-inhibitory impact on its catalytic function in the CaM-regulatory region, thereby acting as a pseudosubstrate to the kinase domain cleft [35]. Another mechanism that controls DAPK1 activation is autophosphorylation at Ser308 in its Ca^2+^/CaM binding region. Dephosphorylation of phosphorylated DAPK1 results in its functional activation [35]. Furthermore, DAPK1 phosphorylation activity contributes to cell necrosis, apoptosis, autophagy, and tumor biology [36–38]. This complexity aligns with the highly intricate etiology of HTN, which includes, but is not limited to, RAAS activation in SHR [39–41]. Consistently, we found that DAPK1 inhibited HBP and improved vascular function in SHRs and Ang II-induced hypertensive mice, suggesting its potential role in anti-HTN. However, the anti-hypertensive role of DAPK1 needs further investigation in other animal models, including two-kidneys-one-clip, deoxycorticosterone acetate, and salt-sensitive models. Additionally, further studies are required to assess the effect of DAPK1 on the resistance index, pulsatility index, vasoconstriction, and vasodilation of resistance vessels, including mesenteric arteries.

These functional studies highlight the essential role of DAPK1 in HTN, encouraging further investigation of its underlying mechanisms. RNA sequencing results demonstrated diverse DETs, including MAPK8 [42], Foxo3 [43], Caspase3 [44], and enriched pathways such as the TNF [45], PI3K-Akt [46], and Jak-STAT pathways [47], all of which play a vital role in HTN-induced vascular dysfunction. These pathways may be DAPK1 targets that reduce hypertensive vascular dysfunction. Furthermore, the integrated analysis of both comparisons revealed significant enrichment in pathways associated with actin cytoskeleton regulation, vascular smooth muscle contraction, and calcium signaling. Excessive vasoconstriction, a key pathological feature of HTN, leads to increased pressure mediated by vascular smooth muscle [48]. As an important bioactive peptide of RAAS, Ang II binds to cell membrane receptors and induces the release of sarcoplasmic reticulum calcium, leading to vasoconstriction [49]. Our results show that DAPK1 knockout ameliorates Ang II-induced vasoconstriction. However, further research should determine the DAPK1 effects on vasoconstriction and vasodilation in response to the activation of other channels: receptor-operated channels, store-operated channels, or voltage-operated calcium channels. Additionally, further studies should assess the effect of DAPK1 on vasoconstriction and vasodilation in small-sized arteries.

Calcium is a crucial second messenger that triggers smooth muscle cell contraction [50–52]. The fast constriction of blood vessels is caused by alterations in the concentration of calcium, which trigger the phosphorylation of the MLC, as well as actin-myosin cross-bridge cycling [20]. The identification of MLC as a DAPK1 substrate has enabled *in vitro* DAPK1 kinase assays, providing a valuable tool for assessing its catalytic activity and regulatory mechanisms [53]. Previous research has shown that DAPK1 can phosphorylate MLC at Ser19 *in vivo*, leading to enhanced actomyosin contractility [54]. Our results indicate that DAPK1 inhibits MLC phosphorylation in the AA of SHRs and Ang II-induced HTN models. Detachment, rounding of the cell, membrane blebbing, shrinkage, and other morphological alterations are observed in several cell types when DAPK1 is forcefully expressed [55,56]. Our objective was to reveal the implication of the Ca^2+^/DAPK1/p-MLC(S19) axis in VSMC cytoskeletal architecture by delving into actin polymerization. Herein, Ang II-induced actin polymerization was reduced by the DAPK1 inhibitor TC-DAPK6. Furthermore, TC-DAPK6 treatment significantly reduced the p-MLC (S19) expression in Ang II-stimulated VSMCs. The outcomes indicate that DAPK1 mitigates the triggering of the MLC signaling pathway, perhaps elucidating how DAPK1 reduces BP and diminishes hypertensive vascular dysfunction. Accordingly, the regulatory role of DAPK1 in other implicated signaling requires further validation in future research.

In brief, DAPK1 plays an essential function in modulating vascular constriction, as well as BP, by modifying the MLC signaling pathway. The DAPK1 /p-MLC (S19) signaling overactivation in VSMCs constitutes a significant innovative mechanism behind Ang II-associated HTN and vascular disorders. Our findings support further investigation into developing DAPK1 inhibitors as potential therapeutic agents for HTN treatment.

**Clinical perspectives**

- Death-associated protein kinase 1 (DAPK1) is a well-known tumor suppressor gene involved in apoptosis, autophagy, and tumor progression. However, its role in hypertension (HTN) remains unclear. Our study aimed to explore whether DAPK1 contributes to HTN pathogenesis.
- Our study demonstrates that DAPK1 inhibition not only lowers blood pressure (BP) but also protects against damage to critical organs such as the heart and kidneys in hypertensive models. Mechanistically, DAPK1 inhibition blocked myosin light chain (MLC) phosphorylation at serine 19, reducing vasoconstriction and protecting against HTN. This suggests that targeting DAPK1 could provide dual benefits in HTN management by simultaneously controlling BP and reducing the risk of end-organ damage.
- This mechanistic understanding provides a foundation for developing therapies that specifically address vascular dysfunction in HTN, potentially leading to more precise and effective treatments for patients with resistant or severe forms of HTN.

## Supplementary material

Online supplementary figure 1

Online supplementary figure 2

Online supplementary figure 3

Online supplementary figure 4

Online supplementary figure 5

Online supplementary figure 6

## Data Availability

Data will be made available on request. RNA sequencing data are available at NCBI GEO (GSE263223).

## Abbreviations

AAV: administered adeno-associated virus
AAs: abdominal aortas
ARRIVE: Animal Research Reporting of In Vivo Experiments
AT1R: angiotensin type 1 receptor
BP: blood pressure
CaM: calmodulin
DAPK1: death-associated protein kinase 1
DBP: diastolic blood pressure
DETs: differential expressed transcripts
FBS: fetal bovine serum
GAPDH: Glyceraldehyde-3-Phosphate Dehydrogenase
GO: Gene Ontology
HBP: high blood pressure
HTN: hypertension
IHC: immunohistochemical
II: calmodulin-dependent kinase II
II: angiotensin II
KD: knocked-down
KEGG: Kyoto Encyclopedia of Genes and Genomes
MAP: mean arterial pressure
MLC: myosin light chain
MMRRC: Mutant Mouse Resource & Research Centers
NIH: National Institutes of Health
PCNA: proliferating cell nuclear antigen
PSS: physiological saline solution
PVDF: polyvinylidene difluoride
PWV: pulse wave velocity
RAAS: renin-angiotensin-aldosterone system
RT: room temperature
SBP: systolic blood pressure
SHRs: spontaneously hypertensive rats
VSMCs: vascular smooth muscle cells
WKY: wistar-Kyoto
