# Supplementary figures and images for: DAPK1 acts as a positive regulator of hypertension via induction of vasoconstriction
Source: Clin Sci (Lond). 2025 Jun 18;139(12):667–81. doi: 10.1042/CS20255840 (PMC12238816; doi:10.1042/CS20255840)

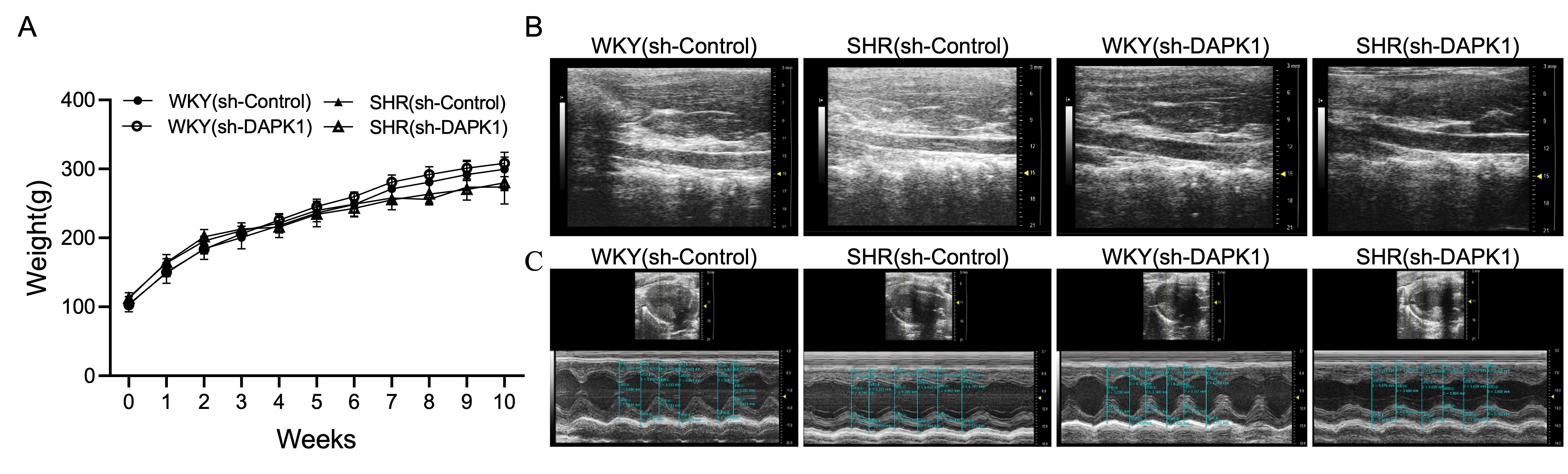

Supplement: Online supplementary figure 1 [file cs-139-12-CS20255840-supp1.jpg]

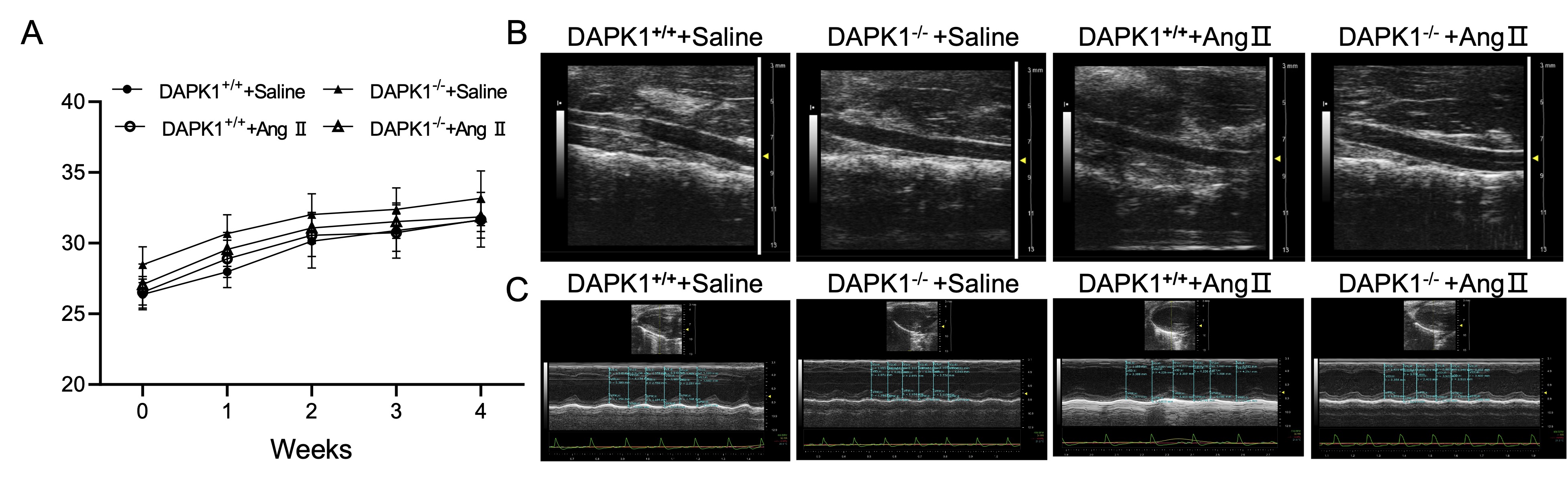

Supplement: Online supplementary figure 2 [file cs-139-12-CS20255840-supp2.jpg]

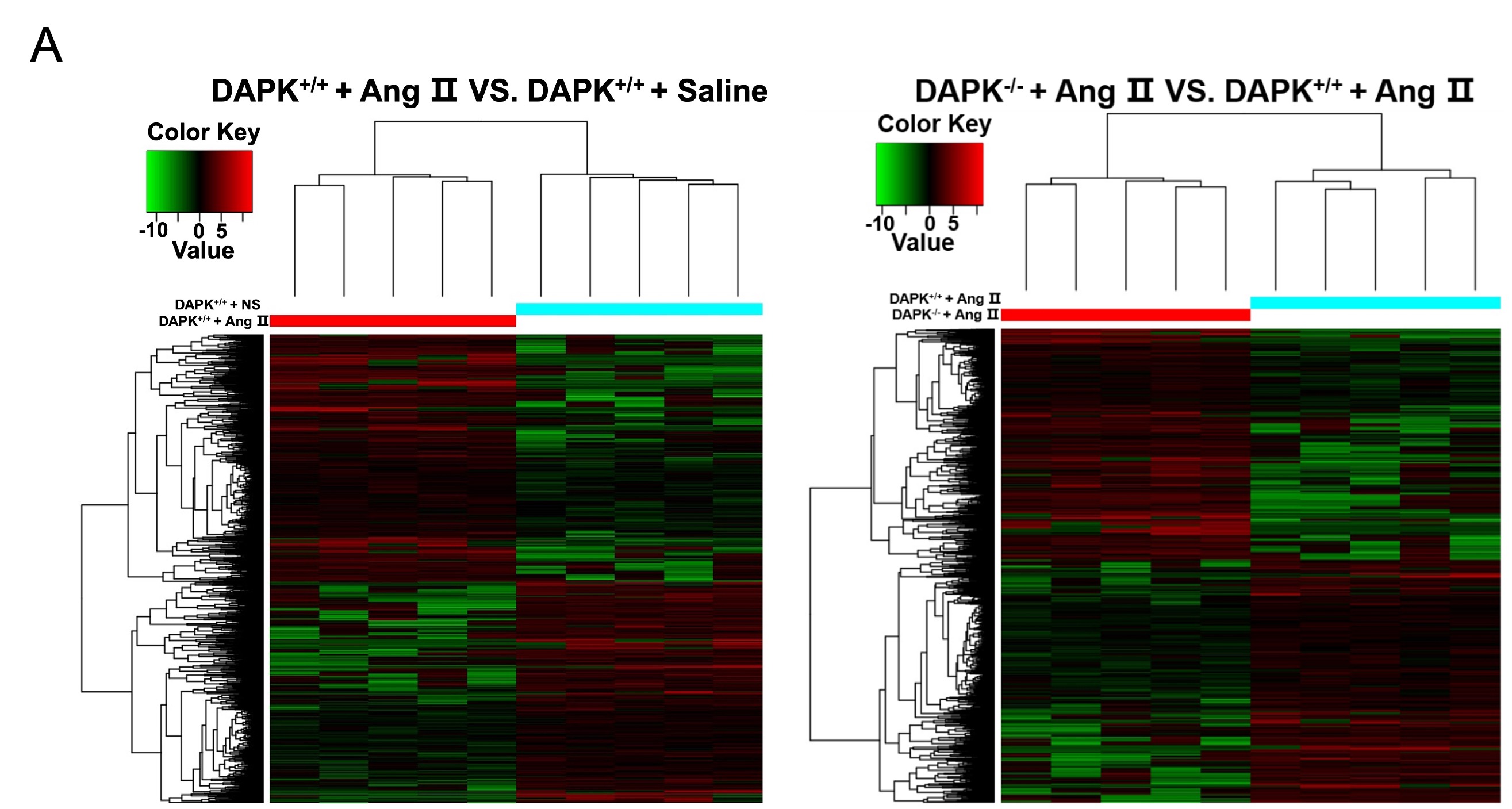

Supplement: Online supplementary figure 3 [file cs-139-12-CS20255840-supp3.jpg]

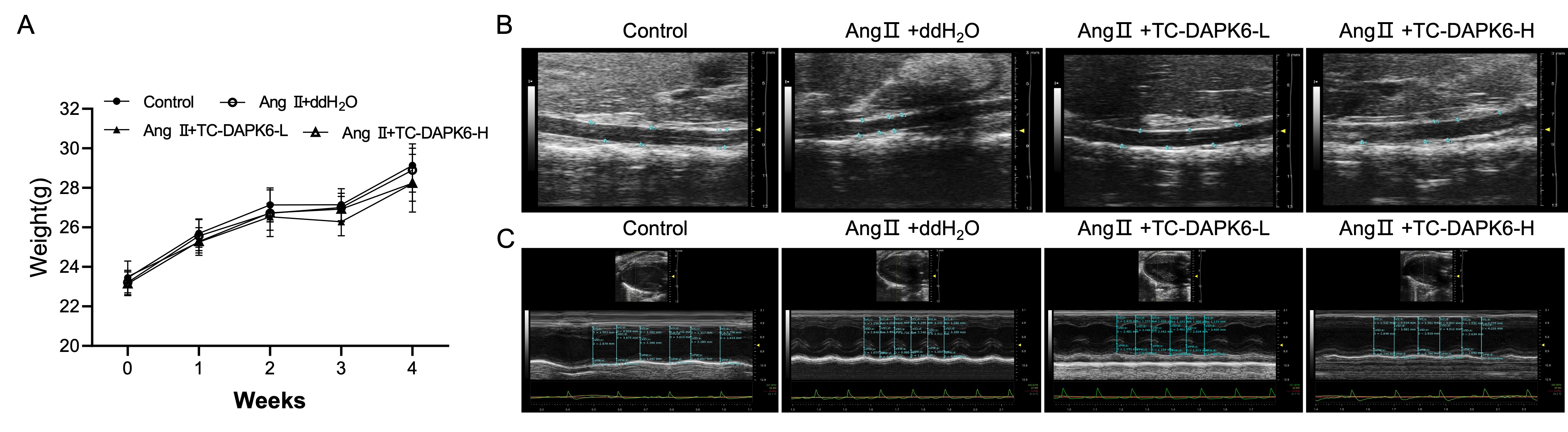

Supplement: Online supplementary figure 4 [file cs-139-12-CS20255840-supp4.jpg]

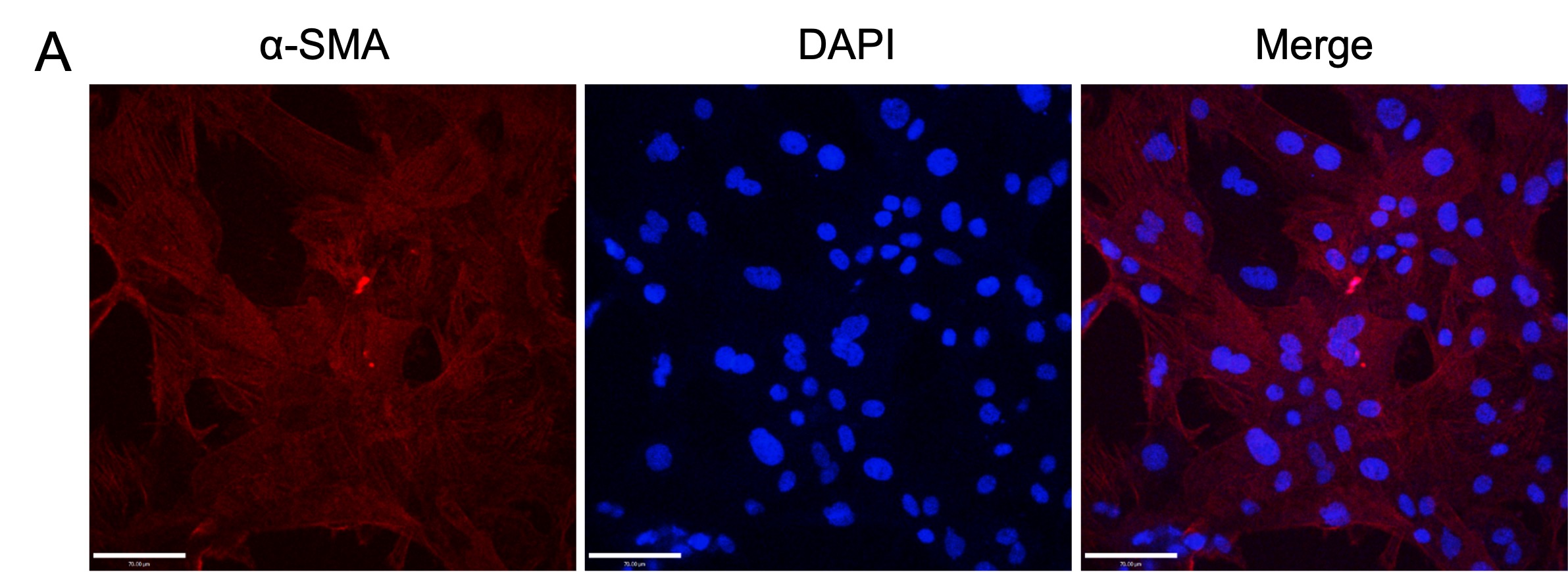

Supplement: Online supplementary figure 5 [file cs-139-12-CS20255840-supp5.jpg]
